# Self-assembly Is Prerequisite for Catalysis of Fe(II) Oxidation by Catalytically Active Subunits of Ferritin*

**DOI:** 10.1074/jbc.M115.678375

**Published:** 2015-09-14

**Authors:** Kourosh Honarmand Ebrahimi, Peter-Leon Hagedoorn, Wilfred R. Hagen

**Affiliations:** From the Department of Biotechnology, Delft University of Technology, 2628 BC Delft, The Netherlands

**Keywords:** enzyme kinetics, ferritin, iron metabolism, isothermal titration calorimetry (ITC), protein self-assembly, enzymology, ferroxidase

## Abstract

Fe(III) storage by ferritin is an essential process of the iron homeostasis machinery. It begins by translocation of Fe(II) from outside the hollow spherical shape structure of the protein, which is formed as the result of self-assembly of 24 subunits, to a di-iron binding site, the ferroxidase center, buried in the middle of each active subunit. The pathway of Fe(II) to the ferroxidase center has remained elusive, and the importance of self-assembly for the functioning of the ferroxidase center has not been investigated. Here we report spectroscopic and metal ion binding studies with a mutant of ferritin from *Pyrococcus furiosus* (PfFtn) in which self-assembly was abolished by a single amino acid substitution. We show that in this mutant metal ion binding to the ferroxidase center and Fe(II) oxidation at this site was obliterated. However, metal ion binding to a conserved third site (site C), which is located in the inner surface of each subunit in the vicinity of the ferroxidase center and is believed to be the path for Fe(II) to the ferroxidase center, was not disrupted. These results are the basis of a new model for Fe(II) translocation to the ferroxidase center: self-assembly creates channels that guide the Fe(II) ions toward the ferroxidase center directly through the protein shell and not via the internal cavity and site C. The results may be of significance for understanding the molecular basis of ferritin-related disorders such as neuroferritinopathy in which the 24-meric structure with 432 symmetry is distorted.

## Teaser

**Background:** Ferritin has 24 subunits that are self-assembled into a spherical shape structure.

**Results:** Blocking self-assembly abolished Fe(II) binding and oxidation in the di-iron catalytic center.

**Conclusion:** Self-assembly is prerequisite for catalysis of Fe(II) oxidation possibly by creating a route for Fe(II) in the protein shell directly toward the catalytic center.

**Significance:** The effect of self-assembly on ferritin functioning is important for understanding ferritin-related disorders.

## Introduction

**FIGURE 1.**
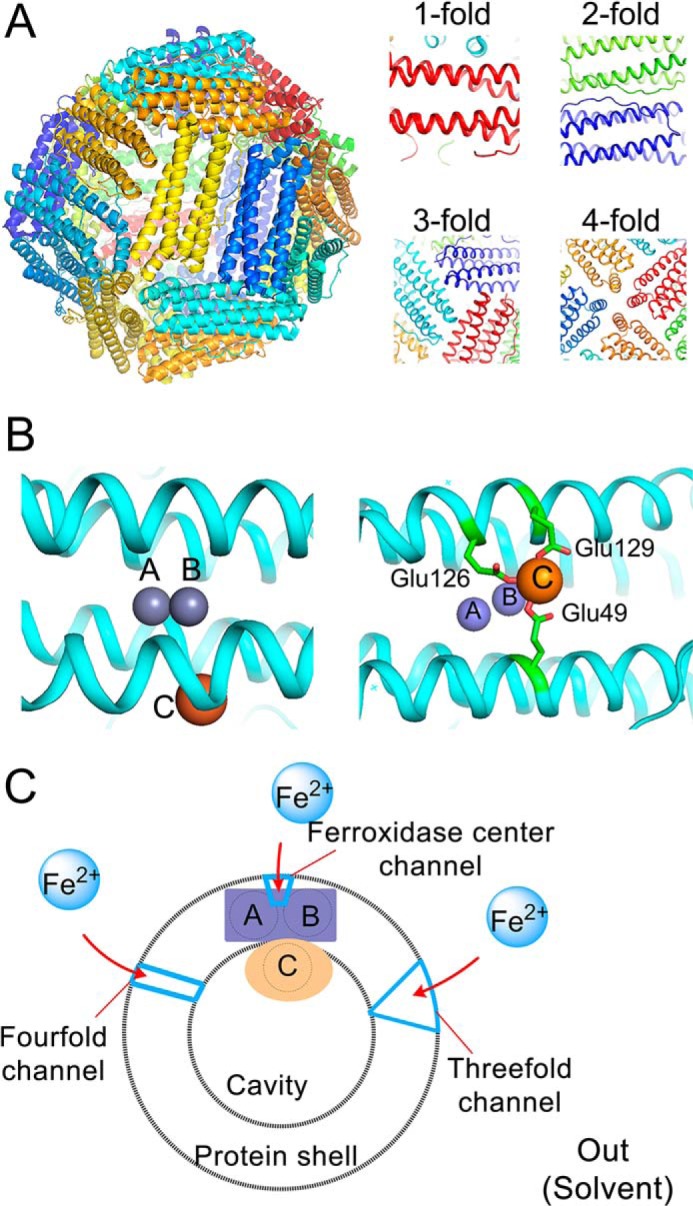
**Structure of ferritin and the location of metal ion binding sites in the ferroxidase center and site C.** *A*, ferritin consists of 24 subunits that form a hollow spherical shape structure with 432 symmetry. *B*, the location of metal ion binding sites in the ferroxidase center and in a third site, site C, near the internal cavity of protein. In PfFtn, the coordinating residues of site C are from two α-helices as observed in the x-ray crystal structure of WT-PfFtn in the presence of Zn(II) (Protein Data Bank code 2JD8). *C*, a cartoon showing the ferroxidase center and site C of one subunit and putative channels: the ferroxidase center channel, the 3-fold channel, and the 4-fold channel.

The ubiquitous iron storage protein of life, ferritin, is a multimeric protein of 24 subunits (Fig. 1*A*), which self-assemble to form a hollow spherical shape structure with 432 symmetry (Fig. 1*A*). The cavity of the protein has a diameter of 8 nm and is surrounded by a protein shell with a thickness of 2 nm. The protein oxidizes Fe(II) to generate Fe(III), which is stored as a ferrihydrite mineral-like particle inside the protein cavity (1–3). Each subunit consists of four α-helices that together form a bundle and of a short C-terminal α-helix. Bacterial and archaeal ferritins have only one subunit type, whereas vertebrate ferritins consist of two or sometimes three different subunit types, which are named based on their relative molecular mass (4–8): L “light” (20 kDa), M “middle” (21.1 kDa), and H “heavy” (22.8 kDa) subunits. In humans, H and L subunits self-assemble with different ratios, which are tissue-dependent, to form heteropolymeric H/L ferritins (4). The H and M subunits of eukaryotic ferritin and all subunits of bacterial or archaeal ferritins have three conserved Fe(II) binding sites (1, 9–11). Two of these sites, denoted A and B (Fig. 1*B*), are located in the middle of the four-α-helical bundle of each subunit, and together are named the ferroxidase center, where catalysis of Fe(II) oxidation occurs. The third site is in the vicinity of the ferroxidase center near the inner surface of the 24-meric protein and is named the gateway site or site C (Fig. 1*B*). Ferritin plays an important role in protecting cells against reactive oxygen species (12, 13), and mutation in L-type ferritin of humans will lead to a neurodegenerative disorder named neuroferritinopathy (14, 15). In addition, ferritin has found applications in different fields (1, 3, 16), for example in biocatalysis (17, 18), in magnetic resonance imaging (19), and in the synthesis of new materials (3, 20–22). Therefore, the fundamental studies regarding its working mechanism are of significant importance. Recent studies have resulted in the proposal of a unifying mechanism for Fe(II) oxidation and storage by eukaryotic, bacterial, and archaeal ferritins (1). It has been observed that Fe(II) distributes among the three metal ion binding sites (23), and as a result in some subunits, two Fe(II) are oxidized in the ferroxidase center, whereas in others three Fe(II) ions are oxidized: two Fe(II) in the ferroxidase center and the third Fe(II) in site C (1, 24, 25). Oxidation of Fe(II) occurs via an intermediate with blue color, which has been observed in archaeal (23, 24), bacterial (25), and eukaryotic ferritins (26), but its precise molecular structure has remained elusive (1). Moreover, in archaeal and eukaryotic ferritin, it has been proposed that when a third Fe(II) is oxidized in site C together with two Fe(II) in the ferroxidase center, a highly conserved tyrosine in the vicinity of site B of the ferroxidase center acts as a molecular capacitor and provides the fourth electron for complete reduction of molecular oxygen to water (24). Subsequently, upon arrival of new Fe(II) ions, the metastable Fe(III) in the ferroxidase center is displaced by Fe(II) ion and only then stored inside the cavity by a yet to be identified mechanism (23, 27). Although significant insight has been obtained regarding the mechanism of Fe(II) oxidation and Fe(III) storage, the effect of self-assembly on catalysis of Fe(II) oxidation at the ferroxidase center and site C has not been studied in detail previously. Furthermore, the exact route of Fe(II) from outside the protein shell to the catalytic center has not been traced out; based on available data for eukaryotic ferritins (28–31) and bacterioferritins (32–36), which are structurally similar to ferritin except for a heme group between pairs of subunits, different pathways may be drawn (1) (Fig. 1*C*).

Here we have studied a ferritin from *Pyrococcus furiosus* (PfFtn)^3^ to explore the possible effect of self-assembly on Fe(II) oxidation at the ferroxidase center and to understand how Fe(II) reaches the ferroxidase center. Using site-directed mutagenesis, we created a mutant with a single amino acid substitution that lacked self-assembly but folded properly and remained thermostable. Subsequent analyses of Fe(II) oxidation kinetics, metal ion binding thermodynamics, and Fe(III) storage in comparison with the wild-type protein provided new insight into the direct impact of self-assembly on Fe(II) entry and binding to the ferroxidase center.

## Experimental Procedures

### 

#### 

##### Chemicals

All chemicals were reagent grade and were purchased from Sigma-Aldrich. Cytochrome *c* from horse heart was also obtained from Sigma-Aldrich.

##### Protein Expression, Purification, and Preparation

WT-PfFtn and its mutant were expressed, purified, and made apo as explained previously (23). A mutant of PfFtn in which arginine 117 was replaced by alanine (R117A-PfFtn) was prepared using a QuikChange^TM^ site-directed mutagenesis kit (Stratagene). For site-directed mutagenesis, the forward primer was: 5′-GAAAAAGATTACTCGACGGCGGCATTCTTAGAGTGG-3′, and the reverse primer was complementary to the forward primer. The insert of interest was sequenced (Baseclear, Leiden, The Netherlands) to confirm the correct mutation.

##### Steady-State Kinetics of Fe(II) Oxidation by WT-PfFtn and R117A-PfFtn

Steady-state kinetics of Fe(II) oxidation were measured by recording the progress curves of Fe(III) formation at 315 nm using a fiber optics spectrophotometer (Avantes) equipped with a homemade thermoblock and cuvette holder. Measurements were performed in 1-ml glass cuvettes with a path length of 1 cm. A molar extinction coefficient of 2.5 mm^−1^ cm^−1^ at 315 nm as reported previously (37) was used to calculated the initial rate of Fe(II) oxidation using the progress curves. Measurements were performed at 37 °C. Buffer was 100 mm Mops, 100 mm NaCl, pH 7.0.

##### Isothermal Titration Calorimetry

A VP-ITC microcalorimeter instrument (Microcal/Malvern) was used to measure Zn(II) binding to WT-PfFtn and R117A-PfFtn. Protein was prepared in 100 mm Mops, 100 mm NaCl, pH 7.0, and the Zn(II) solution was prepared in 100 mm Mops, 100 mm NaCl, pH 6.5, to prevent formation of insoluble Zn(II) hydroxide. The instrument settings were: 30 injections with each injection 3 μl; high feedback mode; interval time, 300 s; injection period, 6 s; stirring speed, 502 rpm; reference power, 15 μcal/s; and temperature, 25 °C. The sample cell was filled with the protein solution, 72 μm (monomer) WT-PfFtn or 102 μm (monomer) R117A-PfFtn, and the syringe was filled with 6.95 mm Zn(II) solution. For each experiment, a control experiment in the absence of protein was performed to obtain the heat of dilution of Zn(II) in buffer. Origin 7.0 software customized by Microcal for analysis of ITC data was used to analyze the data.

##### Dynamic Light Scattering (DLS)

DLS was performed as explained previously (38). Briefly, a Zetasizer NanoZs instrument (Malvern UK) was used to measure the hydrodynamic diameter of ferritin. Measurements were performed at room temperature, 25 °C. Buffer was 100 mm Mops, 100 mm NaCl, pH 7.0. Each measurement consisted of at least 13 runs and was repeated at least two times. Both with WT-PfFtn and R117A-PfFtn four samples were prepared: sample 1, apo protein; sample 2, apo protein aerobically incubated with 2 Fe(II) per monomer; sample 3, apo protein aerobically incubated with 20 Fe(II) per monomer; and sample 4, apo protein aerobically incubated with 50 Fe(II) per monomer. Aerobic addition of Fe(II) was performed stepwise, with each step having two Fe(II) per monomer, to reach the final amount of Fe(II). After each step, the protein was incubated at 50 °C for 5 min.

##### CD Spectroscopy

1 ml of Wt-PfFtn or R117A-PfFtn was washed with at least 200 ml of phosphate buffer (100 mm phosphate, 100 mm NaCl, pH 6.9) using Amicon Ultra filter units with molecular mass cutoff of 10 kDa. This was done to replace Mops buffer, which disturbs the CD spectra of proteins, with phosphate buffer. CD spectra were recorded using a JASCO-815 CD spectrometer as explained previously (38). Measurements were performed at room temperature.

##### Electron Paramagnetic Resonance (EPR) Spectroscopy

Formation of the mixed valence [Fe(II)-Fe(III)] cluster in the ferroxidase center was measured using EPR spectroscopy as explained previously (23). Measurements were performed at 15 K, and the other conditions were as follows: microwave frequency, 9380 MHz; microwave power, 127 milliwatt; modulation frequency, 100 kHz; and modulation amplitude, 12.7 gauss.

##### Mass Spectrometry and Data Analysis

Gel pieces were reduced with dithiothreitol (10 mm), 30 min, room temperature), alkylated with iodoacetamide (20 mm, 60 min, room temperature in the dark), and digested with trypsin overnight at 37 °C. After digestion, formic acid and DMSO were added (both 5% v/v) to increase peptide recovery. Protein digests were analyzed on a reversed phase nano-LC coupled to a LTQ Orbitrap Velos (Thermo Fisher Scientific, Bremen, Germany). An Agilent 1200 series HPLC system was equipped with an in-house packed trapping column (100-μm inner diameter and 20-mm length) and an analytical column (50-μm inner diameter and 250-mm length) filled with Reprosil Pur 120 C18-AQ (Dr. Maisch, Ammerbuch-Entringen, Germany). Trapping was performed at 5 μl/min for 10 min in solvent A (0.1 m acetic acid), and elution was achieved with a gradient from 0 to 40% solvent B (0.1 m acetic acid in 8:2 (v/v) acetonitrile:water) for 40 min. The LTQ Orbitrap Velos was operated in the positive ion mode, using data-dependent acquisition, automatically switching between MS and MS/MS. Full scan MS spectra (from *m*/*z* 400–1500, charge states 2 and higher) were acquired at a resolution of 30,000 at *m*/*z* 400 after accumulation to a target value of 106 ions (automatic gain control). Nine data-dependent MS/MS scans (HCD spectra, resolution 7,500 at *m*/*z* 400) were acquired using the nine most intense ions with a charge state of 2+ or higher and an ion count of 10,000 or higher. MS/MS spectra were analyzed using Proteome Discoverer 1.4 (ThermoFisher Scientific) and Mascot v2.2.02 search engine (Matrix Science). As a reference proteome, the NCBI proteome of *P. furiosus* (2985 sequences) was used. Carbamidomethyl cysteine was set as a fixed modification and oxidized methionine as a variable modification. Trypsin was specified as the proteolytic enzyme, and up to three missed cleavages were accepted. Mass tolerance for fragment ions was set at 0.05 Da and for precursor peptide ions at 10 ppm.

## Results

### 

#### 

##### A Single Amino Acid Substitution at the 3-fold Symmetry Axis Abolished Self-assembly

**FIGURE 2.**
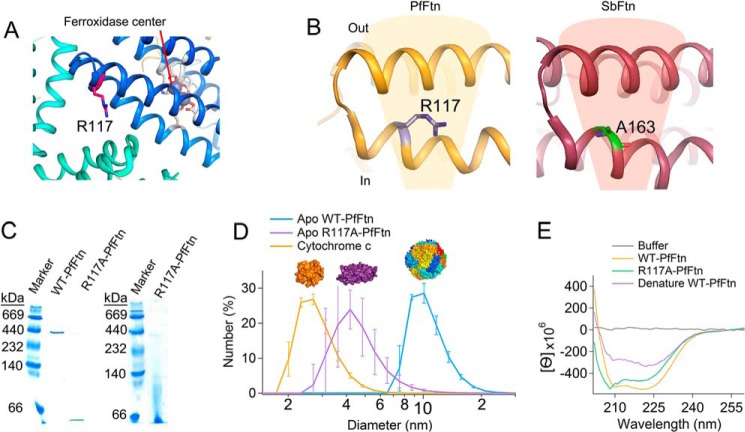
**R117A mutation at the 3-fold symmetry axis abolishes self-assembly.** *A*, the location of Arg-117 in the middle of the 3-fold symmetry axis of PfFtn relative to the ferroxidase center. Arg-117 is more than 16 Å away from the ferroxidase site. *B*, arginine 117 (*R117*) in PfFtn is replaced by an alanine residue (*A163*) in soybean ferritin (*SbFtn*; Protein Data Bank code 3A68). The amino acids in the *shaded area* are located in the putative funnel-like 3-fold channels. *C*, native gel electrophoresis showing the size of WT-PfFtn (5 μm monomer) and R117A-PfFtn (4.3 or 80 μm monomer). *D*, dynamic light scattering was used to measure the hydrodynamic diameter of WT-PfFtn and R117A-PfFtn. Concentration of WT-PfFtn was 25 μm monomer, that of R117A-PfFtn was 32 μm monomer, and that of cytochrome *c* was 260 μm. Measurements were performed at 25 °C, and buffer was 100 mm Mops, 100 mm NaCl, pH 7.0. Data for hydrodynamic diameter of apo R117A-PfFtn are average of 20 measurements ± standard deviation, and other data are average of at least two experiments ± errors. *E*, the CD spectrum of R117A-PfFtn (30 μm monomer) was compared with that of WT-PfFtn (60 μm monomer) and that of WT-PfFtn (60 μm monomer) denatured by addition of ethanol (1/1 ratio).

Based on amino acid substitutions at the 3-fold symmetry axes, where putative channels (3-fold channels) have been proposed to exist, an Fe(II) entry path to the internal cavity of eukaryotic ferritin through 3-fold channels is proposed (29). To test this possibility in PfFtn, we replaced Arg-117 (Fig. 2*A*), which is located in the middle of the 3-fold symmetry axis in the putative path of Fe(II) to the internal cavity and more than 16 Å away from the ferroxidase center, with an alanine (R117A) using site-directed mutagenesis (“Experimental Procedures”). This was done because alanine cannot bind metal ions, and in the x-ray crystal structure of soybean ferritin, an alanine residue (alanine 163) aligns with arginine 117 in PfFtn (Fig. 2*B*). After expression and purification of the protein, to check for formation of stable 24-meric structures, the protein was run on a native gel, and the result was compared with that of wild-type PfFtn (Fig. 2*C*). Although the wild-type protein forms a single band at ∼480 kDa attesting to the presence of stable 24-meric structures, for two different amounts of R117A-PfFtn loaded on the gel essentially a single band at an apparent molecular mass of less than 66 kDa is observed (Fig. 2*C*). Mass spectrometry confirmed that this band is produced by R117A-PfFtn. Thus, it appears that R117A mutation abolished self-assembly and resulted in formation of monomeric (∼20 kDa) and/or dimeric (∼40 kDa) species. Observation of monomeric/dimeric species using gel electrophoresis might have resulted from the possible instability of the quaternary structure of R117A mutant during electrophoretic separation. To exclude this possibility, we used DLS to study the oligomeric state of R117A-PfFtn in solution. DLS was used to measure the hydrodynamic diameter of apo R117A-PfFtn (Fig. 2*D*), and the results were compared with two standards: apo WT-PfFtn with diameter of 11–12 nm and cytochrome *c* with diameter of 3.0 nm (Fig. 2*D*). The hydrodynamic diameter of WT-PfFtn was 10–11 nm, and that of cytochrome *c* was 2.6 nm. For apo R117A-PfFtn, a peak was observed, whose maximum varied for different measurements between 3 and 6 nm. We took the average of 20 measurements, which gave a single peak with a maximum at ∼4.2 nm. This value is close to the theoretical value of 4.3 nm for the hydrodynamic diameter of a monomer. This theoretical value was predicted using the formula for the hydrodynamic diameter of a monomeric subunit assuming a prolate ellipsoid shape (like a rugby ball) (39), with major axes of ellipsoid being the width and the length of a ferritin monomer, *i.e.* 3.6 and 5.8 nm, respectively. If R117A-PfFtn would have consisted of a mixture of monomers (∼3.6-nm width and 6-nm length) and dimers (∼7.2-nm width and 6-nm length), during DLS measurements two maxima should have been observed. Therefore, based on the results of gel electrophoresis and DLS together, we conclude that apo R117A-PfFtn was essentially in the monomeric state.

##### R117A-PfFtn Has a Four-α-Helical Bundle Structure

Using CD spectroscopy, we tested whether R117A-PfFtn folded into a four-α-helical bundle structure (Fig. 2*E*). The CD spectrum of R117A-PfFtn showed characteristics of a protein with a secondary structure that is predominantly α-helical, which is indicated by two negative peaks at ∼222 and 208 nm (Fig. 2*D*). Moreover, comparison of the CD spectrum of R117A-PfFtn with those of proteins or peptides with a four-α-helical bundle structure (40–43) indicated that the tertiary structure of R117A-PfFtn indeed consists of four α-helices that together form a bundle. To confirm this, we unfolded WT-PfFtn by addition of ethanol, and the CD spectrum of the unfolded protein was recorded (Fig. 2*E*). It can be seen that in the unfolded protein, the maximum at 208 nm almost completely diminishes. Subsequently, we checked whether the CD spectrum of R117A-PfFtn had any difference with that of WT-PfFtn. We observed that the CD spectrum of WT-PfFtn was slightly different (Fig. 2*E*). In WT-PfFtn, the peak at ∼222 nm was more intense compared with the peak at 208 nm, whereas in R117A-PfFtn, the peak at 208 nm was the more intense one. A somewhat similar difference has been observed between the dimers and monomers of a 14-kDa protein (ROP (repressor of primer)) with a four-α-helical bundle structure the same as that of subunits of ferritin (44). It has been observed that the interaction between the subunits of ROP changes the CD spectrum of a subunit. Therefore, it appears that the interaction between subunits of ferritin will result in changes in each subunit, which affect the CD spectrum of a subunit. We conclude that R117A-PfFtn folded correctly into a four-α-helical bundle structure.

##### Self-assembly Is Essential for Fe(II) Oxidation in the Ferroxidase Center

**FIGURE 3.**
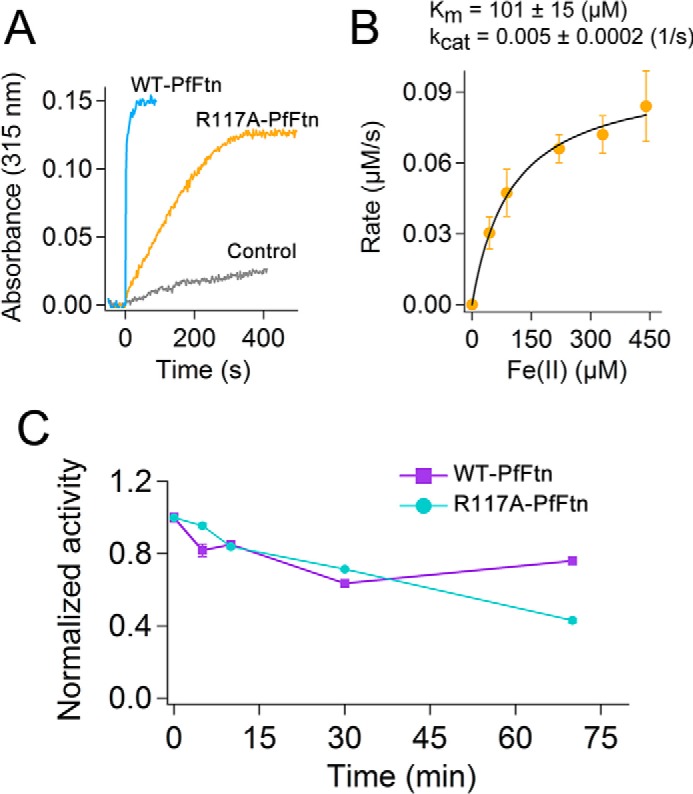
**R117A mutation abolishes fast catalysis of Fe(II) oxidation in the ferroxidase center.** *A*, the progress curve for oxidation of Fe(II) by R117A-PfFtn (22 μm monomer) was compared with that of oxidation of Fe(II) by WT-PfFtn (20 μm monomer) and that of background oxidation of Fe(II) by molecular oxygen in the absence of protein. Progress curves were recorded at 315 nm for formation of Fe(III) after aerobic addition of two Fe(II) per monomer. *B*, kinetics of Fe(II) oxidation by R117A-PfFtn does not show positive cooperativity. The initial rates for formation of Fe(III) were obtained from the progress curves at 315 nm, and the results were plotted as a function of Fe(II)-added per monomer. The *line* shows a fit to the Michaelis-Menten equation. Concentration of R117A-PfFtn was 22 μm monomer. *C*, R117A-PfFtn is thermostable. R117A-PfFtn or WT-PfFtn were incubated in water bath at 100 °C, and samples were taken before the incubation (*t* = 0) and at 5, 10, 30, and 70 min after incubation. Fe(II) oxidation activity was measured by the addition of 5 μl Fe(II) solution (17.2 mm) to a final volume of 1 ml. For each protein, the activity was normalized relative to its maximum activity before boiling (*t* = 0), and the data were plotted as a function of time. The concentration of R117A-PfFtn was 21.5 μm (monomer), and that of WT-PfFtn was 2.1 μm (monomer). The measurements were performed at 37 °C, and the buffer was 100 mm Mops, 100 mm NaCl, pH 7.0. The data are the averages of three experiments ± standard deviation.

We created a mutation, R117A, that abolished self-assembly affording predominantly monomers with four-α-helical bundle structure. As a result this mutation led to the exposure of site C (normally in the inner-surface of the 24-meric protein) to the solvent. This gave us the opportunity to directly investigate the importance of Fe(II) entry into the ferritin cavity and the role of site C as a route for Fe(II) to the ferroxidase center, which is proposed based on studies with bullfrog M-type ferritin (29, 45). We first tested whether the R117A mutation affected the catalysis of Fe(II) oxidation. Fe(II) oxidation was measured after addition of two Fe(II) per subunit, and formation of Fe(III) was followed by recording the absorbance at 315 nm. A control experiment in the absence of protein was performed to measure the rate of background oxidation of Fe(II) by molecular oxygen (Fig. 3*A*). The results showed that the R117A mutation reduced the rate of Fe(II) oxidation more than 100-fold. This is comparable with the more than 100-fold decrease in the rate of Fe(II) oxidation that we observed previously for a single amino acid substitution in site A or B of the ferroxidase center of PfFtn (23), *i.e.* E17H and E130H. Comparison of the rate of Fe(II) oxidation by R117A mutant with that of the nonenzymatic background oxidation of Fe(II) by molecular oxygen indicated that this mutant ferritin was still able to oxidize Fe(II) at a significantly faster rate compared with the background oxidation of Fe(II) by molecular oxygen. Subsequently, we checked whether kinetics of Fe(II) oxidation by R117A mutant exhibits positive cooperativity as has been reported previously for WT-PfFtn (37), and whether it proceeds via the blue intermediate observed for Fe(II) oxidation by WT-PfFtn (23, 24). We recorded the formation of Fe(III) product at 315 nm after addition of different amounts of Fe(II) per monomer. Then the initial rates of formation of Fe(III) were plotted as a function of Fe(II) added per monomer (Fig. 3*B*). The results fitted well to the Michaelis-Menten equation and showed no evidence of positive cooperativity. Furthermore, in the R117A mutant formation of the blue intermediate in the ferroxidase center during catalysis of Fe(II) oxidation was not observed. The inability of R117A-PfFtn to catalyze fast oxidation of Fe(II) might have been resulted from heating the protein at 85 °C for 10 min during purification. To test this possibility, we measured the thermostability of R117A-PfFtn compared with WT-PfFtn. Apo R117A-PfFtn or apo WT-PfFtn were boiled at 100 °C, and samples were taken at 0, 5, 10, 30, or 70 min to measure Fe(II) oxidation activity of the proteins (Fig. 3*C*). Both WT-PfFtn and R117A-PfFtn showed high thermostability up to ∼30 min. After 70 min, WT-PfFtn lost ∼30% of its activity, whereas R117A-PfFtn lost ∼50% of its activity. Thus, it appears that after 30 min R117A-PfFtn is less thermostable than WT-PfFtn, which is presumably related to extra stabilization in the 24-meric structure of WT-PfFtn. Thus, we conclude that the R117A mutation abolished oxidation of Fe(II) by the ferroxidase center and that the monomers were not affected by the heat step purification.

##### Self-assembly Is Essential for Fe(II) Binding to the Ferroxidase Center but Not to Site C

**FIGURE 4.**
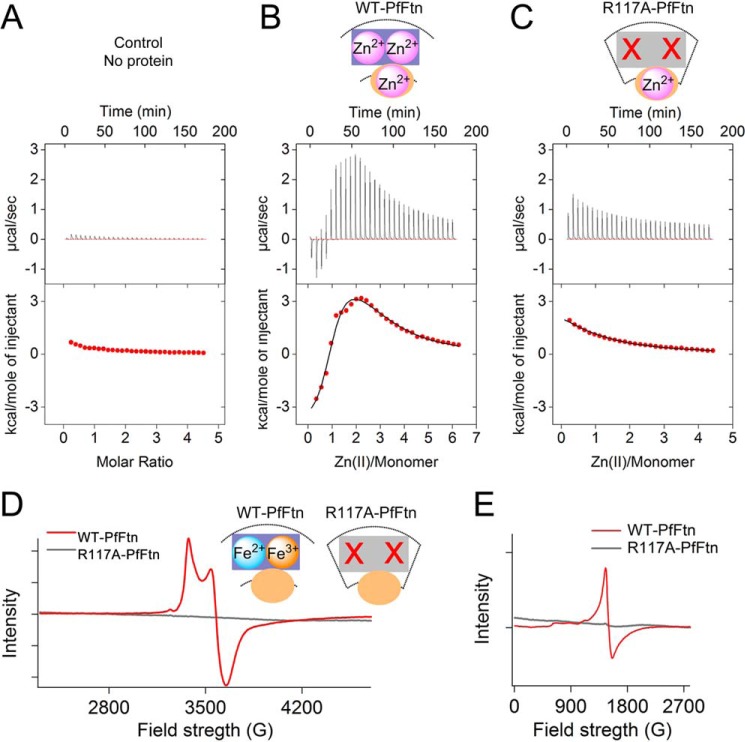
**R117A mutation abolishes metal ion binding to the ferroxidase center but not to site C.** *A*, heat of dilution of Zn(II) in buffer. *B* and *C*, Zn(II) binding to WT-PfFtn (72 μm monomer, *B*) and to R117A-PfFtn (102 μm monomer, *C*) as measured by ITC. The results for WT-PfFtn were fitted using a model of three-binding sites, and the results for R117-PfFtn were fitted using a model of one binding site. Measurements were performed at 25 °C. The buffer was 100 mm Mops, 100 mm NaCl, pH 7.0. The data in *B* and *C* are corrected for the heat of dilution of Zn(II) in buffer in the absence of protein. *D*, Fe(II) binding to the ferroxidase center as recorded by EPR spectroscopy. Fe(II) binds to the ferroxidase center of WT-PfFtn and is oxidized. Subsequently, the metastable Fe(III) is displaced by newly added Fe(II), and a mixed valence [Fe(II)-Fe(III)] cluster is formed in the ferroxidase center. In R117A-PfFtn, no mixed valence signal was observed. *E*, EPR spectra of mononuclear Fe(III) species in WT-PfFtn and R117A-PfFtn. For EPR experiments, the concentration of WT-PfFtn or that of R117A-PfFtn was 250 μm monomer.

To understand why R117A-PfFtn mutant lacked the fast catalysis of Fe(II) oxidation, we examined metal ion binding to apo WT-PfFtn and apo R117-PfFtn. Zn(II) binding to apo R117A-PfFtn was measured using ITC, and the results were compared with those of Zn(II) binding to apo WT-PfFtn. A control experiment in the absence of protein was performed to obtain the heat of dilution of Zn(II) in buffer (Fig. 4*A*). The results for apo WT-PfFtn showed the best fit to a model of three sequential binding sites each with stoichiometry of one (Fig. 4*B*). The thermodynamic parameters for these sites are given in Table 1. Comparison of the results with those obtained for Fe(II) binding to sites A, B, and C in WT-PfFtn (23) suggests that Zn(II) binds to the same sites as Fe(II). These results are also consistent with the observation of three Zn(II) binding sites, two sites in the ferroxidase center and site C, in the x-ray crystal structure of PfFtn (11). The R117A mutation abolished Zn(II) binding to two of the sites, and the data could be fitted using a model of one binding site (Fig. 4*C*). The association constant and the thermodynamic properties of this site were close to those of site C in WT-PfFtn (Table 1). Thus, it appears that R117A mutation abolished binding of Zn(II) to the ferroxidase center but not to site C. The x-ray crystallography (Fig. 1*B*) shows that coordinating residues of Zn(II) in site C are from two α-helices (11). Therefore, the observation that site C in R117A-PfFtn can bind Zn(II) is consistent with the results of CD spectroscopy suggesting folding of R117A-PfFtn into a four-α-helical bundle structure. Subsequently, we investigated binding of Fe(II) to the ferroxidase center using EPR spectroscopy. We have previously shown that anaerobic addition of Fe(II) to Fe(III)-loaded WT-PfFtn or WT-HuHF, which have Fe(III) in the ferroxidase center, will lead to displacement of Fe(III) in the ferroxidase center by Fe(II) and result in the formation of mixed valence [Fe(II)-Fe(III)] cluster in the ferroxidase center (23), which can be recorded using EPR spectroscopy. Thus, first two Fe(II) per subunit were added aerobically to apo WT-PfFtn to fill the ferroxidase centers with Fe(III). After complete oxidation of Fe(II), two Fe(II) were added anaerobically, and formation of mixed valence [Fe(II)-Fe(III)] cluster in the ferroxidase center was recorded using EPR spectroscopy (Fig. 4*D*). As observed previously, the addition of Fe(II) to the Fe(III)-loaded WT-PfFtn resulted in displacement of Fe(III) in the ferroxidase center by Fe(II) and formation of mixed valence [Fe(II)-Fe(III)] cluster (Fig. 4*D*). However, R117A-PfFtn was EPR silent, and no mixed valence Fe(II)-Fe(III) species (Fig. 4*D*) or Fe(III) species (Fig. 4*E*) was observed. Therefore, it appears that Fe(II) binding to the ferroxidase center was fully abolished because of R117A mutation.

**TABLE 1:** **Thermodynamic parameters of Zn(II) binding to apo Wt-PfFtn and apo R117A-PfFtn** Zn(II) binding to apo WT-PfFtn and R117A-PfFtn was measured using isothermal titration calorimetry. Concentration of Wt-PfFtn was 72 μm (monomer), and that of R117A-PfFtn was 102 μm (monomer). Measurements were performed at 25 °C. Buffer was 100 mm Mops containing 100 mm NaCl, pH 7.0. The data for Fe(II) binding to apo WT-PfFtn are from Ref. 23.

Parameters	Apo WT-PfFtn	Apo R117A-PfFtn	Apo WT-PfFtn
	*Zn(II)*	*Zn(II)*	*Fe(II)*
*N*_A_	1		1
*K*_A_ (m^−1^)	(2.56 ± 0.4) × 10^5^		(2.4 ± 0.2) × 10^6^
Δ*H*_A_ (kJ/mol)	−14.1 ± 0.8		−19.9 ± 0.2
*N*_B_	1		1
*K*_B_ (m^−1^)	(1.99 ± 0.5) × 10^4^		(7.9 ± 0.3) × 10^4^
Δ*H*_B_ (kJ/mol)	+38.5 ± 4.7		+32.2 ± 0.5
*N*_C_	1	0.9 ± 0.1	1
*K*_C_ (m^−1^)	(8.5 ± 1.5) × 10^3^	(7.8 ± 0.8) × 10^3^	(1.7 ± 0.4) × 10^4^
Δ*H*_C_ (kJ/mol)	+15.5 ± 4.5	+20.5 ± 2.6	+28.7 ± 1.5

##### Site C Catalyzes Slow Oxidation of Fe(II) in the R117A Mutant

**FIGURE 5.**
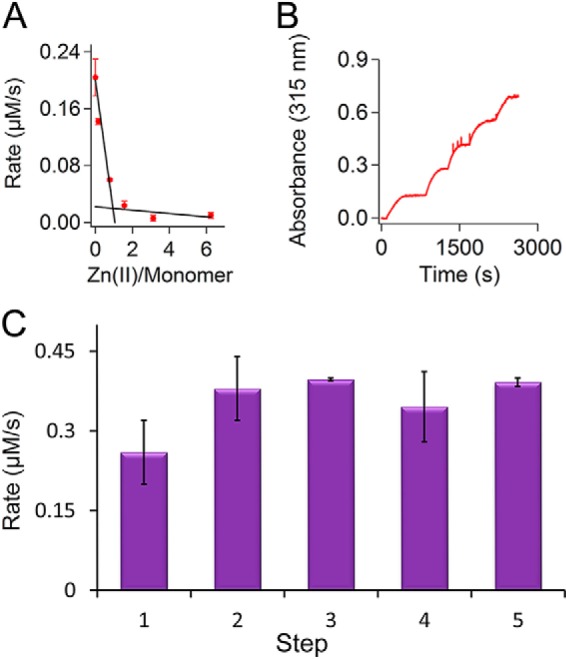
**Site C catalyzes slow oxidation of Fe(II) in R117A mutant.** *A*, Zn(II) binds site C and inhibits oxidation of Fe(II) by R117A-PfFtn. The initial rate of Fe(II) oxidation (four Fe(II) per monomer) in the presence of different amounts of Zn(II) was determined from the progress curves for formation of Fe(III) at 315 nm. The initial rates were plotted as a function of Zn(II) per monomer. In the presence of ∼1 Zn(II) per monomer, oxidation of Fe(II) is completely abolished. Concentration of R117A-PfFtn was 16 μm monomer. Measurements were performed at 37 °C. *B*, R117A-PfFtn can oxidize Fe(II) in subsequent steps. Fe(II) was added in five steps, with each step having two Fe(II) per monomer, to R117A-PfFtn (22 μm monomer), and progress curves for formation of Fe(III) were recorded at 315 nm. *C*, from the progress curves the initial rate of Fe(II) oxidation for each step is obtained. Measurements were performed at 37 °C. The data are averages of three experiments ± standard deviation.

We observed that although Fe(II) did not bind to the ferroxidase center in the R117A mutant, this mutant could still oxidize Fe(II) with a significantly higher rate compared with the background oxidation of Fe(II) by molecular oxygen in the absence of protein. To understand how the R117A mutant can oxidize Fe(II), we tested whether site C was involved in this relatively slow catalysis of Fe(II) oxidation. To this goal, we measured Fe(II) oxidation kinetics in the presence of Zn(II), which is a known inhibitor of Fe(II) oxidation by eukaryotic, bacterial, and archaeal ferritins (46–48). Four Fe(II) per monomer were added to apo R117A-PfFtn in the presence of different amounts of Zn(II) per monomer, *i.e.* 0, 0.2, 0.8, 1.6, 3.1, or 6.3 Zn(II) per monomer, and progress curves for the formation of Fe(III) species were measured. The initial rate of Fe(III) formation was determined using the initial slope of the progress curves and the molar extinction coefficient of the Fe(III) mineral particle in PfFtn at 315 nm (“Experimental Procedures”). The resulting initial rates as a function of Zn(II) per monomer were plotted (Fig. 5*A*). From these results, we observed that at approximately one Zn(II) per monomer, the slow catalysis of Fe(II) oxidation was almost completely abolished. Because the results of ITC suggest that site C in R117A-PfFtn can bind Zn(II), the results of Fe(II) oxidation in the presence of different amounts of Zn(II) suggest that site C is involved in binding to Fe(II) and its slow oxidation by R117A-PfFtn. This is consistent with the observation that the rate of Fe(II) oxidation in R117A-PfFtn is comparable with the rate of Fe(II) oxidation by the ferroxidase center mutants of PfFtn, in which site C is present. Then we tested whether the Fe(III) formed in site C as a result of Fe(II) oxidation remains in this site or spontaneously moves from this site. If Fe(III) would stay at site C, we expected a higher rate of Fe(II) oxidation in apo R117A-PfFtn than in Fe(III)-loaded R117A-PfFtn. This is in analogy to the observation that in Fe(III)-loaded WT-PfFtn occupancy of the ferroxidase center with Fe(III) reduces the rate of catalysis of Fe(II) oxidation compared with apo WT-PfFtn (37). To test this possibility, we measured oxidation of Fe(II) by R117A-PfFtn in five steps (Fig. 5*B*). The initial rates of Fe(III) formation (Fig. 5*C*) for all the steps were within the experimental error the same. Thus, in R117A-PfFtn Fe(II) is oxidized with a slow rate at site C, and the Fe(III) product leaves this site and moves to the site where Fe(III) nucleation begins. This is consistent with the EPR measurement of Fe(III) species in R117A-PfFtn, which did not give any signal for mononuclear Fe(III) species (Fig. 4*E*).

##### R117A Mutant Stores Fe(III) but Cannot Control the Size of the Fe(III) Mineral Particle

**FIGURE 6.**
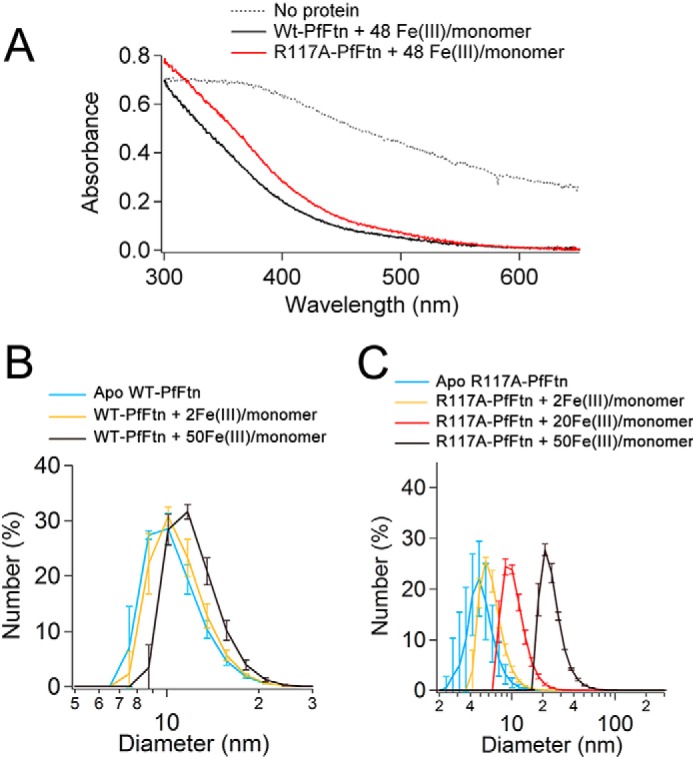
**R117A-PfFtn can store Fe(III).** *A*, the UV-visible spectrum of Fe(III) mineral in WT-PfFtn is compared with that of Fe(III) mineral in R117A-PfFtn and to the spectrum of insoluble Fe(III) in the absence of protein. Spectra were recorded after aerobic addition of Fe(II) in 12 steps, with each step having 4 Fe(II) per monomer in the presence or absence of protein. Concentration of WT-PfFtn and of R117A-PfFtn was 5 μm (monomer). Measurements were performed at 50 °C. *B* and *C*, dynamic light scattering measurements of WT-PfFtn (*B*) and of R117A-PfFtn (*C*) before and after aerobic addition of Fe(II) to load ferritin with different amounts of Fe(III) per monomer. Concentration of WT-PfFtn and that of R117A-PfFtn was 25 μm. The measurements were performed at 25 °C. The data are averages of two experiments ± errors.

We then tested whether the R117A mutant was able to store the Fe(III) in a soluble form. Aerobic addition of 2, 10, or 40 Fe(II) per subunit to R117A-PfFtn (200 μm monomer) did not lead to the precipitation of Fe(III). Thus, we compared the absorbance of Fe(III) mineral in R117A-PfFtn with that in WT-PfFtn. After aerobic addition of 48 Fe(II) per monomer in 12 steps, each step four Fe(II) per monomer and incubation time of 5 min, we recorded the absorbance of the Fe(III) mineral particle for WT-PfFtn and for R117A-PfFtn, and the results were compared with the absorbance of Fe(III) in the absence of protein (Fig. 6*A*). It can be observed that the absorbance spectrum of the Fe(III) species formed by WT-PfFtn and by R117A-PfFtn is the same, which suggests that a similar Fe(III) mineral species was formed as a result of Fe(II) oxidation by both proteins. On the other hand, in the absence of protein scattering of light caused by formation of insoluble Fe(III) mineral led to an increase in absorbance at all wavelengths. To understand how the monomeric R117A-PfFtn was able to store Fe(III) in a soluble form, we used dynamic light scattering to determine the oligomeric state of R117A-PfFtn after addition of Fe(II) and formation of Fe(III). The results were compared with those for WT-PfFtn. Both R117A-PfFtn and WT-PfFtn were aerobically incubated with different amounts of Fe(II), *i.e.* 2, 20, or 50 Fe(II) per monomer as explained under “Experimental Procedures.” DLS measurements showed that the hydrodynamic diameter of apo WT-PfFtn increased from 10–11 to 12–13 nm upon loading the protein with 50 Fe(III) per monomer (Fig. 6*B*). This suggests that the protein expanded ∼1 nm and controls the size of Fe(III) mineral core. For R117A-PfFtn, however, after loading the protein with 2 Fe(III) per monomer, a peak at 6 nm was observed, which is close to the hydrodynamic diameter of dimers with a width of ∼7.0 nm and length of ∼6.0 nm (Fig. 6*C*). Increasing the amount of Fe(III) further to 20 Fe(III) per monomer resulted in formation of larger oligomeric structures with a peak at a hydrodynamic diameter of 10–11 nm. Further aerobic addition of Fe(II) to load the protein with up to 50 Fe(III) per monomer resulted in formation of oligomeric state(s) with a peak at 25 nm (Fig. 6*C*). Therefore, it appears that R117A-PfFtn is able to catalyze Fe(III) mineral formation but lacks the ability to control the size of the Fe(III) mineral particle because of its inability to form a stable 24-meric structure. These results are consistent with the results of CD spectroscopy. If R117A-PfFtn were misfolded, it would have been unable to form a soluble complex with mineral Fe(III), and Fe(III) should have precipitated.

## Discussion

Ferritin self-assembles from 24 subunits, and the catalytically active subunits oxidize Fe(II) for Fe(III) storage. Previous studies with 24-meric eukaryotic ferritin have led to the suggestion that Fe(II) needs to enter the protein cavity from where it reaches the ferroxidase center via a conserved site C (29, 45). This proposal implies that if the self-assembly is abolished and stable monomers are formed, site C, which will be exposed to the solvent, will allow Fe(II) entry to the ferroxidase center. The effect of self-assembly on the functioning of the ferroxidase center and site C has never been reported before; in a previous study only the effect of denaturation/renaturation of the 24-meric structure of a mutant of HuHF on Fe(II) oxidation activity was studied (49). A mutant of HuHF, *i.e.* L169R, which self-assembled to a 24-meric structure, was denatured in the presence of 6 m guanidine HCl (pH 3.0) and subsequently renatured by increasing the pH to 7.0. This denaturation/renaturation process led to formation of monomers. Fe(II) oxidation activity measurements before denaturation and after renaturation were compared with that of background and not with that of wild-type HuHF. Based on these data, it was concluded that the presence of fully assembled 24-meric cage is important for the Fe(II) oxidation activity. However, the rates of Fe(II) oxidation reported for L169R-HuHF before denaturation of 24-mers and after renaturation were only slightly higher than that of background oxidation of Fe(II) by molecular oxygen but were significantly (at least 100-fold) lower than that of Fe(II) oxidation by wild-type HuHF, which was reported in a previous study (50). Therefore, the decrease in the rate of Fe(II) oxidation by L169R-HuHF appears to be mainly due to L169R mutation and not to the denaturation/renaturation process. Based on these data, we cannot obtain insight regarding the effect of self-assembly on Fe(II) binding to the ferroxidase center and its subsequent oxidation at this site. Therefore, we studied a mutant of PfFtn in which self-assembly was abolished by a single amino acid substitution and monomers were formed directly and not by denaturation of 24-meric cage. This allowed us to study the significance of self-assembly for Fe(II) binding to the ferroxidase center of individual monomers and its subsequent oxidation at this site and to obtain new insight regarding the role of the conserved site C in Fe(II) entry to and/or Fe(III) exit from the ferroxidase center. Stable monomers of PfFtn, which folded into a four-α-helical structure, were obtained as a result of a single amino acid substitution (R117A) in the 3-fold symmetry axis. Fe(II) oxidation activity measurements and metal ion binding studies showed that Fe(II) or Zn(II) could not bind to the ferroxidase center. Zn(II) was able to bind to site C. The affinity and thermodynamic properties of Zn(II) binding to site C in R117A-PfFtn were within experimental error the same as those observed for Zn(II) binding to the site C in WT-PfFtn. Binding of Zn(II) to site C inhibited the relatively slow oxidation of Fe(II) by R117-PfFtn, which suggests that Fe(II) was able to bind to site C and was oxidized at this site. Because of these results and because R117 is more than 16 Å away from the ferroxidase center (Fig. 1*B*), we do not have any reason to believe that the R117A mutation directly affected the amino acid residues near site C and the ferroxidase center, or the stability and folding of the protein. Instead R117A mutation should have affected Fe(II) binding and oxidation at the ferroxidase center and lower thermostability of R117A-PfFtn compared with WT-PfFtn by abolishing the self-assembly. These results appear to be not in favor of a model in which Fe(II) needs to enter the internal cavity of protein and from there reaches the ferroxidase center via individual metal ion binding sites in the internal surface of ferritin including site C. In this light, we re-evaluated three recent studies based on which a route of Fe(II) to the ferroxidase center through the internal cavity and via the conserved site C was defined (29, 45, 51).

Site-directed mutagenesis of two amino acid residues at the 3-fold symmetry axis, E130A and D127A, of bullfrog M ferritin (BfMF) was shown to abolish formation of the blue intermediate in this protein (29), from which it has been concluded that these residues are involved in Fe(II) translocation to the internal cavity of protein from where Fe(II) reaches the ferroxidase center via the conserved site C (29). A similar conclusion was derived in a more recent study in which a single amino acid substitution in the 3-fold symmetry axis of BfMF, *i.e.* D127E, abolished the rate of Fe(II) oxidation in the ferroxidase center (51). However, in both studies it has not been checked whether in BfMF mutation of the residues at 3-fold symmetry axis affects assembly of the protein, possibly resulting in inability of the ferroxidase center to properly bind the Fe(II) ions and thus to abolishment of Fe(II) oxidation in the ferroxidase center as here observed in PfFtn. In fact for the D127E mutant of BfMF, x-ray crystallography showed only monomers (51) and not the self-assembled 24-meric structure reported for wild-type BfMF (52). In a separate study using x-ray crystallography, the molecular structure of WT-BfMF was solved after aerobic and anaerobic incubation with Fe(II) for different time intervals (45). Under aerobic conditions, the electron density of metal ion was observed in the putative 3- and 4-fold channels, at the ferroxidase center, and at site C, but under anaerobic conditions, the electron density for Fe(II) was not observed in the 3-fold channel. The electron density of metal ion in the putative 3-fold channels under aerobic conditions was assigned to Fe(II), and based on this, a path of Fe(II) entry was defined as follow: Fe(II) enters the ferritin cavity via the eight 3-fold channels and then enters the ferroxidase center via site C. However, the electron density for metal ion in the putative 3-fold channels for the crystals prepared under aerobic conditions (45) cannot reasonably be associated with Fe(II) but instead should be related to Fe(III). Therefore, based on the x-ray crystallographic study of BfMF aerobically incubated with Fe(II) one cannot draw the conclusion that in BfMF the putative 3-fold channel (3-fold pore) is a path for Fe(II) entry into the ferritin cavity from where it would then reach site C and subsequently the ferroxidase center. When crystals of ferritin from Pseudo-nitzschia multiseries were grown anaerobically in the presence of Fe(II), also no electron density for Fe(II) ion was observed in the putative 3-fold channels and/or other putative channels to the internal cavity (53). It is possible that Fe(II) enters via other routes and distributes among Fe(II) binding sites in the ferroxidase center and site C.

**FIGURE 7.**
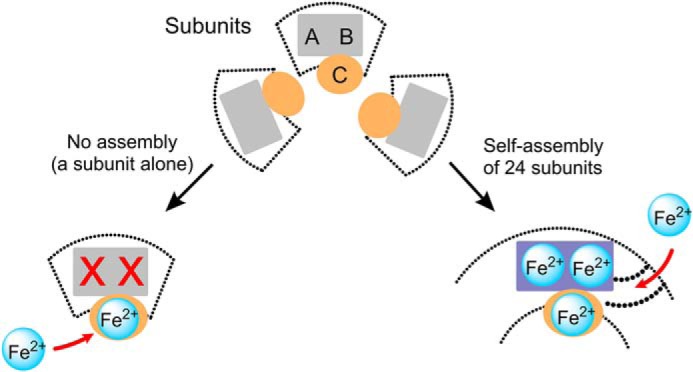
**A new model for Fe(II) entry into ferritin.** Self-assembly creates a direct route for Fe(II) to the ferroxidase center through the protein shell. In this model, site C is not a route for Fe(II) to the ferroxidase center; instead, it functions as a gateway for the ferroxidase center through which the metastable Fe(III) in the ferroxidase center moves toward the internal cavity of the protein for Fe(III) mineral formation.

In conclusion, although the exact path of Fe(II) remains to be identified, our data are in favor of a new model for Fe(II) translocation to the ferroxidase center (Fig. 7): self-assembly leads to conformational changes in the four α-helix bundle of each subunit forming channels that guide the Fe(II) directly toward the ferroxidase center and site C through the protein shell (Fig. 7), resulting in distribution of Fe(II) among the three binding sites as demonstrated previously (23). In this model, site C in the internal cavity is not involved in Fe(II) translocation to the ferroxidase center. Previously we have suggested that site C might act as a gateway for Fe(II) entry to the ferroxidase center and/or Fe(III) exit from the ferroxidase center (1, 23). Based on our present studies, we now propose that site C is possibly a gateway for Fe(III) in the ferroxidase center to enter the internal cavity of protein. Although the results strongly support this model, we cannot exclude the possibility that self-assembly induces conformational changes in the amino acid residues of the ferroxidase center enabling the ferroxidase center to bind Fe(II). Future studies, *e.g.* using x-ray crystallography together with site-directed mutagenesis, should provide further evidence regarding the exact effect of self-assembly on Fe(II) entry and/or Fe(II) binding to the ferroxidase center, which may reveal the precise function of site C. Nevertheless our study demonstrates that self-assembly is a key determinant of Fe(II) binding to the ferroxidase center and its subsequent oxidation by molecular oxygen. This new finding might also have implications for the iron-homeostasis process and ferritin-related disorders. For example, in humans the progressive neurodegenerative disorder neuroferritinopathy has been shown to be caused by a mutation at the C terminus of the L subunit in heteropolymeric H/L ferritin (15). This mutation affects self-assembly of H and L subunits of ferritin (54). As a consequence, it might affect catalysis of Fe(II) oxidation in the ferroxidase center at pH values at which Fe(II) ions bind to the ferroxidase center, *i.e.* pH ≥ 7.0 (55), but not at lower pH values, *e.g.* pH 6.5 at which Fe(II) binding to the ferroxidase center is abolished (55).

## Author Contributions

K. H. E. designed and performed the experiments. K. H. E., P.-L. H., and W. R. H. analyzed data and wrote the manuscript.

## Footnotes

3 The abbreviations used are:

PfFtn: *P. furiosus* ferritin
DLS: dynamic light scattering
EPR: electron paramagnetic resonance
BfMF: bullfrog M ferritin
ITC: isothermal titration calorimetry.
